# Genome-wide analysis of the ATP-binding cassette (ABC) transporter gene family in sea lamprey and Japanese lamprey

**DOI:** 10.1186/s12864-015-1677-z

**Published:** 2015-06-06

**Authors:** Jianfeng Ren, Yu-Wen Chung-Davidson, Chu-Yin Yeh, Camille Scott, Titus Brown, Weiming Li

**Affiliations:** Key Laboratory of Exploration and Utilization of Aquatic Genetic Resources, College of Fisheries and Life Sciences, Shanghai Ocean University, Shanghai, 201306 China; Department of Fisheries and Wildlife, Michigan State University, East Lansing, MI 48824 USA; Department of Computer Science and Engineering, Michigan State University, East Lansing, MI 48824 USA; Department of Microbiology and Molecular Genetics, Michigan State University, East Lansing, MI 48824 USA

**Keywords:** ABC transporter, Evolution, Chordates, Lampreys, RNA-Seq, Gene expression

## Abstract

**Background:**

Lampreys are extant representatives of the jawless vertebrate lineage that diverged from jawed vertebrates around 500 million years ago. Lamprey genomes contain information crucial for understanding the evolution of gene families in vertebrates. The ATP-binding cassette (ABC) gene family is found from prokaryotes to eukaryotes. The recent availability of two lamprey draft genomes from sea lamprey *Petromyzon marinus* and Japanese lamprey *Lethenteron japonicum* presents an opportunity to infer early evolutionary events of ABC genes in vertebrates.

**Results:**

We conducted a genome-wide survey of the ABC gene family in two lamprey draft genomes. A total of 37 ABC transporters were identified and classified into seven subfamilies; namely seven ABCA genes, 10 ABCB genes, 10 ABCC genes, three ABCD genes, one ABCE gene, three ABCF genes, and three ABCG genes. The ABCA subfamily has expanded from three genes in sea squirts, seven and nine in lampreys and zebrafish, to 13 and 16 in human and mouse. Conversely, the multiple copies of *ABCB1*-, *ABCG1*-, and *ABCG2*-like genes found in sea squirts have contracted in the other species examined. *ABCB2* and *ABCB3* seem to be new additions in gnathostomes (not in sea squirts or lampreys), which coincides with the emergence of the gnathostome-specific adaptive immune system. All the genes in the ABCD, ABCE and ABCF subfamilies were conserved and had undergone limited duplication and loss events. In the sea lamprey transcriptomes, the ABCE and ABCF gene subfamilies were ubiquitously and highly expressed in all tissues while the members in other gene subfamilies were differentially expressed.

**Conclusions:**

Thirteen more lamprey ABC transporter genes were identified in this study compared with a previous study. By concatenating the same gene sequences from the two lampreys, more full length sequences were obtained, which significantly improved both the assignment of gene names and the phylogenetic trees compared with a previous analysis using partial sequences. The ABC gene subfamilies in chordates have undergone obvious expansion or contraction. The ABCA subfamily showed the highest gene expansion rate during chordate evolution. The evolution of ABC transporters in lampreys requires further evaluation because the present results are based on a draft genome.

**Electronic supplementary material:**

The online version of this article (doi:10.1186/s12864-015-1677-z) contains supplementary material, which is available to authorized users.

## Background

The ATP-binding cassette (ABC) gene family encodes membrane-spanning proteins that transport a wide variety of substrates (e.g., ions, sugars, amino acids, lipids, lipopolysaccharides, peptides, metals, toxic metabolites, and xenobiotics) across cell membranes. ABC transporters are ubiquitous in all organisms from prokaryotes to eukaryotes [1–4]. Most ABC proteins contain an ATP-binding domain (also known as the nucleotide-binding domain, NBD) and a transmembrane domain (TMD). The highly conserved NBD contains Walker A, Walker B, and ABC signature motifs. The NBD binds and hydrolyses ATP, providing energy for substrate transport. The TMD consists of five to six membrane spanning helices that determine substrate specificity [5]. Eukaryotic ABC transporters are either full transporters with all the required domains in one polypeptide chain (i.e., two NBDs and two TMDs), or half transporters (one NBD and one TMD) that form homo- or heterodimers as the functional units [1–4].

ABC proteins have been divided into eight distinct subfamilies (A–H) based on the features of their NBDs. Subfamily H, which is closely related to subfamily G, was first identified in fruit fly (*Drosophila melanogaster*) and later found in insects and zebrafish (*Danio rerio*), but is absent from plant, worm, yeast, and mammalian genomes [6, 7]. The remaining seven subfamilies (A–G) have been named ABC1, MDR/TAP, CFTR/MRP, ALD, OABP, GCN20, and White, respectively [1, 8]. ABC transporters can also be classified functionally as exporters, importers, and non-transport proteins. The exporters and importers are essential for transporting diverse biological substances, whereas the non-transport proteins (subfamilies E and F) are involved mainly in ribosome biogenesis and translation regulation [9–11].

Many ABC transporters associated with disease processes have been studied extensively in animal models. ABCB1 (P-glycoprotein) was the first eukaryotic ABC transporter to be identified on the surface of cancer cells [12] where it acts as a multidrug resistance (MDR) efflux transporter that prevents the accumulation of chemotherapeutic drugs inside cancer cells. Some ABC genes have been associated with hereditary diseases such as cystic fibrosis caused by mutations in ABCC7/CFTR (cystic fibrosis transmembrane conductance regulator), adrenoleukodystrophy (ALD) caused by mutations in ABCD1, and cholesterol metabolism disorders caused by mutations in ABCB4, ABCB11, and ABCC2 [13].

The ABC transporter family with a total of 48 members was first systematically characterized in human (*Homo sapiens*) [1]. The ABC transporter genes in mouse (*Mus musculus*) and fruit fly genomes have been analyzed jointly with their counterparts in the human genome [1, 14]. Subsequently, genome-wide analyses of ABC genes have been performed in the nematode *Caenorhabditis elegans* [15], several arthropod species including mosquito (*Anopheles gambiae*) [16], silkworm (*Bombyx mori*), the beetle *Tribolium castaneum*, honeybee (*Apis mellifera*) [17–19], water flea (*Daphnia pulex*) [20], spider mite (*Tetranychus urticae*) [21], and several fish species including the channel catfish *Ictalurus punctatus* [22].

The evolution of the ABC transporter genes has been investigated within the jawed vertebrate lineage (gnathostome) [7, 13, 23], but not in the jawless vertebrate lineage (lampreys and hagfishes; agnatha). Jawless vertebrates diverge from the jawed vertebrates near 500 million years ago [24], and differ from gnathostomes by a single medially-located nostril and a notochord that persists in adults. Jawless vertebrates also lack hinged jaws, a mineralized skeleton, paired appendages, a pancreas, and spleen [25].

The draft genomes of two lamprey species, the sea lamprey *Petromyzon marinus* and Japanese lamprey *Lethenteron japonicum*, have been released recently [24, 26]. These genome data provide resources for investigating ABC gene evolution in early vertebrates. In this study, we conducted a genome-wide survey of the ABC gene family in sea lamprey and Japanese lamprey, and identified 37 ABC transporters. Phylogenetic analyses divided these ABC genes into seven subfamilies. We also obtained the ABC gene expression levels in various tissues across different developmental stages in sea lamprey by high-throughput RNA sequencing (RNA-Seq). The patterns of ABC gene expansion or contraction were discussed in the context of chordate evolution.

## Results and discussion

### Identification, characterization, and phylogenetic analysis of ABC transporters

The sea lamprey genome is characterized by having many chromosomes (n is about 99), and is highly repetitive, heterozygous, and GC-rich [27]. The genome was found to undergo programmed genome rearrangements during early embryogenesis, resulting in the deletion of about 20 % of the germline DNA from somatic tissues [28]. Thus, the sea lamprey genome sequences that have been published are more fragmented and cover fewer genes than the genome encodes. Therefore, we assembled a sea lamprey transcriptome from 89 RNA-Seq samples of various tissues across different developmental stages (Additional file 1: Figure S1) that greatly improved the percentage of genes covered. In addition, a draft genome assembly of the Japanese lamprey has been generated recently with DNA from the testis [26], thereby avoiding the DNA deletion that takes place in somatic tissues.

The ABC genes were identified independently in the sea lamprey and Japanese lamprey genomes. These sea lamprey and Japanese lamprey ABC gene sequences have been deposited in GenBank and are presented in the Additional file 2, respectively. A total of 37 ABC transporter genes were identified in the lampreys and 13 more ABC transporter genes were identified in this study compared with the previous analysis in Liu et al [22] (Additional file 3: Table S1). The lengths of mRNA and protein sequences, CDS status, domain structure, and GenBank accession numbers are summarized in Table 1. The phylogenetic analyses grouped the 37 transporter genes into seven subfamilies (Additional file 4: Figure S2): namely, seven ABCAs, 10 ABCBs, 10 ABCCs, three ABCDs, one ABCE, three ABCFs, and three ABCGs (Table1). The ABCH subfamily member that was present in zebrafish was not found in the lamprey genomes. In the phylogenetic analyses, the sea squirts *Ciona intestinalis* and *C. savignyi* that represent a non-vertebrate chordate lineage were chosen as the outgroups. Detailed evolutionary and phylogenetic analyses of the ABC genes in lampreys were conducted for each subfamily as described below.

**Table 1:** Details of 37 ABC transporter genes and the translated proteins identified in lampreys

Gene name	Sea lamprey (SL)				Japanese lamprey (JL)			Concatenation of SL and JL				Reference^b^ (aa)
	Acc. No.	mRNA (bp)	Protein (aa)	CDS status^a^	mRNA (bp)	Protein (aa)	CDS status	mRNA (bp)	Protein (aa)	Domain structure	CDS status	
ABCA1a	KM232912	4371	1457	P	6588	2195	C	6786	2276	(TMD-NBD)2	C	2261
ABCA1b	KM232913	609	203	P	4416	1471	P	4416	1471	TMD-NBD-TMD	P	2261
ABCA2	KM232914	4614	1537	P	7053	2350	C	7521	2457	(TMD-NBD)2	C	2433
ABCA3	KM232915	7248	1725	C	4455	1484	C	7248	1725	(TMD-NBD)2	C	1704
ABCA4	KM232916	3180	1059	P	6348	2116	C	6348	2116	(TMD-NBD)2	C	2310
ABCA5	KM232917	5366	1679	C	3762	1253	P	5366	1679	(TMD-NBD)2	C	1642
ABCA12	KM232918	6737	1832	P	5492	1829	P	6737	1832	(TMD-NBD)2	P	2595
ABCB1	KM232919	4585	1288	C	3750	1249	C	4585	1288	(TMD-NBD)2	C	1276
ABCB1-like	KM232920	2256	751	P	3018	1005	P	3132	1043	TMD-?-TMD-NBD	P	1276
ABCB5	-	-	-	-	3570	1189	C	3570	1336	(TMD-NBD)2	C	1255
ABCB6	KM232921	4180	857	C	2382	793	C	4180	857	TMD-NBD	C	842
ABCB7	KM232922	2241	729	C	-	-	-	2242	729	TMD-NBD	C	752
ABCB8	KM232923	2371	709	C	2118	705	C	2371	709	TMD-NBD	C	717
ABCB9	KM232924	3063	807	C	2385	795	C	3063	807	TMD-NBD	C	762
ABCB10	KM232925	3635	754	C	2265	754	C	3635	754	TMD-NBD	C	715
ABCB10-like	KM232926	2370	790	C	2259	752	C	2370	790	TMD-NBD	C	715
ABCB11	KM232927	5241	1320	C	2853	950	P	5241	1320	(TMD-NBD)2	C	1321
ABCC1	KM232928	1484	444	P	4044	1347	P	4658	1501	(TMD-NBD)2	C	1528
ABCC2	KM232929	7002	1558	C	4034	1344	P	7002	1558	(TMD-NBD)2	C	1543
ABCC3a	KM232930	5876	1556	C	4260	1419	P	5876	1556	(TMD-NBD)2	C	1522
ABCC3b	KM232931	3500	1166	P	2859	952	P	3502	1166	(TMD-NBD)2	P	1522
ABCC4	KM232932	4176	1303	C	2070	689	P	4176	1303	(TMD-NBD)2	C	1325
ABCC5	KM232933	3093	1009	P	4236	1411	C	4383	1439	(TMD-NBD)2	C	1436
ABCC7	KM232934	4606	1466	C	3180	1059	P	4606	1466	(TMD-NBD)2	C	1476
ABCC8	KM232935	4737	1578	C	4311	1436	P	4737	1578	(TMD-NBD)2	C	1588
ABCC9	KM232936	4850	1596	C	4383	1460	P	4850	1596	(TMD-NBD)2	C	1588
ABCC10	KM232937	5922	1584	C	4710	1569	C	5922	1584	(TMD-NBD)2	C	1460
ABCD2	KM232938	2954	791	C	2379	792	C	2954	791	TMD-NBD	C	741
ABCD3	KM232939	1990	659	C	1980	659	C	1990	659	TMD-NBD	C	659
ABCD4	KM232940	2033	633	C	1902	633	C	2033	633	TMD-NBD	C	606
ABCE1	KM232941	2386	599	C	1800	599	C	2386	599	NBD-NBD	C	599
ABCF1	KM232942	2773	740	C	2508	835	C	3037	828	NBD-NBD	C	837
ABCF2	KM232943	5349	614	C	1845	614	C	5349	614	NBD-NBD	C	628
ABCF3	KM232944	3774	712	C	2022	673	C	3774	712	NBD-NBD	C	709
ABCG2a	KM232945	2790	703	C	2100	699	C	2790	703	NBD-TMD	C	657
ABCG2b	KM232946	2521	666	C	1737	578	C	2521	666	NBD-TMD	C	657
ABCG4	KM232947	1977	658	C	1959	652	C	1977	658	NBD-TMD	C	646

^a^P indicates partial sequence, C indicates complete sequence. ^b^Reference indicates the length of the protein in mouse

### ABCA subfamily

**Fig. 1:**
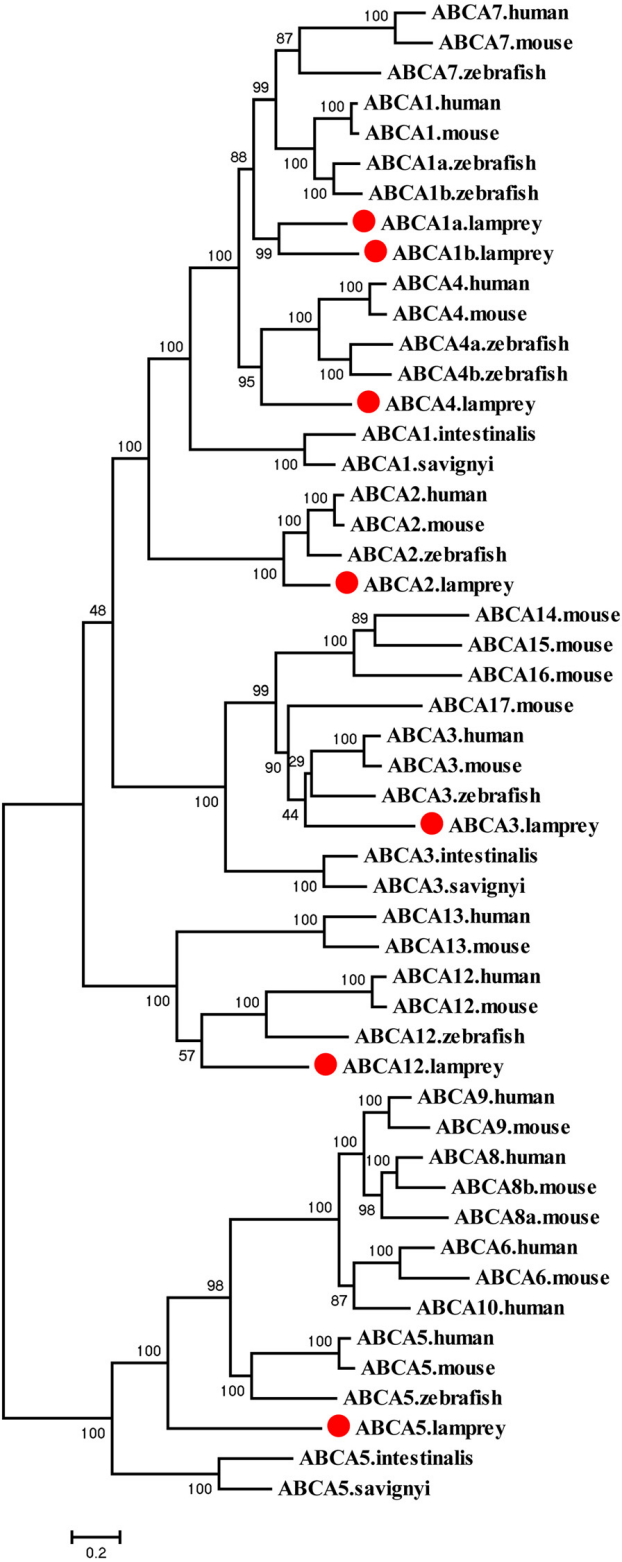
Phylogenetic tree of ABCA subfamily transporters. Amino acid sequences were aligned using ClustalX 1.83 and the multiple alignment was used to construct the phylogenetic tree using MEGA6 with 100 bootstrap replicates. The phylogenetic tree was constructed using a maximum likelihood method with the Jones–Taylor–Thornton (JTT) substitution model. The lamprey ABCA proteins are highlighted with red dots

Seven ABCA genes were identified in the sea lamprey and Japanese lamprey including *ABCA1a*, *ABCA1b*, *ABCA2*–*ABCA5*, and *ABCA12*. These genes all encode full transporter proteins, except ABCA1b, which lacks a NBD because of incomplete sequence (Table 1, Additional file 5: Figure S3). *ABCA1b* and *ABCA4* were not found in the previous study by Liu et al [22] and most of the identified ABCA genes were short fragments and less than half their full length (Additional file 3: Table S1). The phylogenetic analysis supported the member assignment for the lamprey ABCA subfamily and the seven ABCA proteins clustered with their counterparts from other species (Fig. 1).

The lamprey ABCA1a and ABCA1b proteins clustered with members of the ABCA1 and ABCA7 from human, mouse and zebrafish. This large clade then clustered with the ABCA4 group with sea squirt ABCA1 members at the base of the whole clade (Fig. 1). The ABCA7 and ABCA1 members from gnathostomes appear as paralogs derived from ABCA1 duplication because two ABCA1 members from the lampreys were at the base of the clade. Two copies of both ABCA1 and ABCA4 encoded in zebrafish may be derived from fish-specific genome duplication because they are also present in most teleost fish [22]. ABCA2 appears before the speciation of lampreys and is generally conserved in vertebrates, but has not been found in some teleost fish examined [22].

Sea squirt ABCA3 proteins were placed at the basal position of the clade including the vertebrate ABCA3 group, and the mouse ABCA14–ABCA16 group, and ABCA17 (Fig. 1). This finding implies that mouse *ABCA14*–*ABCA17* may be derived from the duplication of *ABCA3*. The gene arrangement analysis also suggested that they evolved by gene duplication. In the mouse genome, the first exons of *ABCA17* and *ABCA3* overlap and are transcribed from opposite strands [29]. *ABCA14*–*ABCA16* are arranged in a tandem head-to-tail cluster on a mouse chromosome and this arrangement is also present in the rat and dog genomes but only the gene fragments of *ABCA14*–*ABCA16* remain in human genome [7, 30]. *ABCA12* is present in the genomes of all four vertebrates examined (human, mouse, zebrafishand lamprey) while *ABCA13* is present only in the human and mouse genomes (Fig. 1).

*ABCA5* is conserved throughout all chordates, as indicated by the phylogenetic analysis. In a large clade, the ABCA5 proteins of sea squirts and lamprey were placed at the base whereas the ABCA5s of human, mouse, and zebrafish clustered together in a subclade that included ABCA6, ABCA8, and ABCA9 proteins from both mouse and human, as well as a ABCA10 from human (Fig. 1). No counterparts for the ABCA5-related genes (*ABCA6* and *ABCA8*–*ABCA10*) were identified in lamprey, zebrafish, or other teleost fish [22]. These ABCA5-related genes in the human, dog (*ABCA10* is a pseudogene), and mouse (*ABCA8* is duplicated and *ABCA10* is absent) were arranged in a cluster with *ABCA5* on the same chromosomes [7, 31]. Other ABCA5-related genes that clustered with *ABCA5* have also been found on chicken and *Xenopus* chromosomes [23, 31]. The phylogenetic and syntenic analyses imply that *ABCA5* is evolutionarily conserved and the additions of *ABCA5*-related genes occurred and expanded through gene duplication after the teleost split with the other vertebrates [32].

In summary, most of the ABCA genes identified in this study were full length after concatenation of the two lamprey sequences and two more ABCA genes were identified than Liu et al [22]. Sea squirts have three members in the ABCA subfamily whereas lamprey and zebrafish have seven and nine, respectively. However, mouse and human possess up to 16 and 13 members, respectively. This finding clearly implies that the ABCA subfamily has undergone expansion through high-frequency gene duplication during chordate evolution.

### ABCB subfamily

**Fig. 2:**
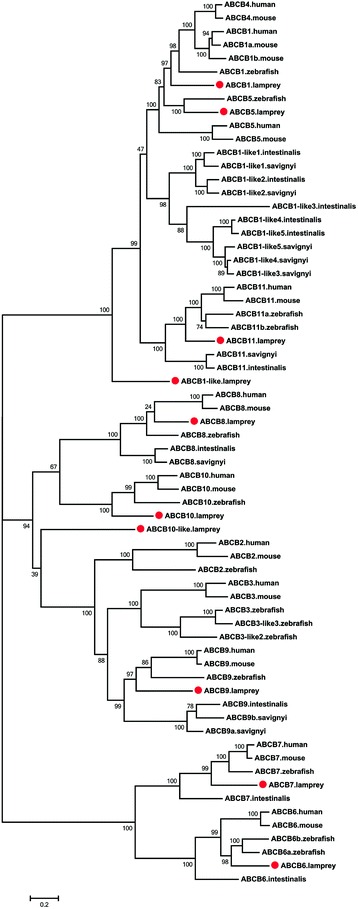
Phylogenetic tree of ABCB subfamily transporters. The phylogenetic tree was constructed as described in the legend to Fig. 1. The lamprey ABCB proteins are highlighted with red dots

A total of 10 ABCB genes were identified in lampreys including *ABCB1*, *ABCB5*–*ABCB11*, *ABCB1*-like, and *ABCB10*-like. Four of these genes, *ABCB5*, *ABCB7*, *ABCB1*-like, and *ABCB10*-like were not found by Liu et al [22] and most of the gene sequences were shorter than the full length gene sequences identified in the present study. Furthermore, in the previous study *ABCB1* was fragmented into two segments that were annotated mistakenly as “*ABCB4*” and “*ABCB5*” (Additional file 3: Table S1). The ABCB subfamily includes both full and half transporters. The ABCB1, ABCB5, and ABCB11 proteins are full transporters while ABCB1-like is an incomplete sequence that lacks a NBD. The remaining proteins, ABCB6–ABCB10 and ABCB10-like are half transporters (Table 1, Additional file 5: Figure S3). Compared with the human genome with 11 ABCB transporter genes, lampreys lacked ABCB2–ABCB4. However, two additional genes, *ABCB1*-like and *ABCB10*-like were identified in lampreys (Fig. 2).

As shown in the phylogenetic tree (Fig. 2), ABCB4 is absent in sea squirts, lampreys, and zebrafish. In addition, the lamprey ABCB1 and ABCB5 proteins fell into a large subclade that included the ABCB1, ABCB4, and ABCB5 proteins from human, mouse, and zebrafish. This large subclade then grouped with the subclade of five predicted ABCB1-like proteins from sea squirts. This finding implies that the *ABCB1*-like genes in sea squirts underwent significant gene loss such that one member remained after the divergence of lampreys with sea squirts or that *ABCB1*-like underwent *Ciona*-specific duplications. Gene duplications also occurred and gave rise to new ABCB members such as *ABCB4* and *ABCB5. ABCB1* and *ABCB4* in the human and mouse genomes are co-orthologous to *ABCB1* in the fish genomes, which was confirmed by a genomic syntenic analysis that showed that *ABCB4* occurred by duplication of *ABCB1* in mammals, estimated to be 170 million years ago, after the split of mammals from birds and reptiles [22]. An additional study also showed that *ABCB1*, *ABCB4*, and *ABCB5* were closely related and shared a common ancestor in chordate history [33]. *ABCB5* was thought to be specific to mammalian genomes [23], but was later discovered in non-mammalian genomes including teleost, birds, lizards, and *Xenopus* [22]. Here, we found that *ABCB5* appeared much earlier in the vertebrate lineage, in the lampreys.

*ABCB6*–*ABCB9* and *ABCB11* are more conserved than other members of the ABCB subfamily in chordate, as indicated by their presence in genomes from sea squirt to human. Furthermore, fish-specific genome duplication and species-specific duplication may have led to the emergence of *ABCB6b* and *ABCB11b* respectively in zebrafish (Fig. 2), as determined from an examination of *ABCB6* and *ABCB11* in teleost fish genome [22]. The ABCB2 (also known as antigen peptide transporter 1, TAP1) and ABCB3/TAP2 were not found in either sea squirts or lampreys, but are present in zebrafish and species that evolved later. The ABCB2 and ABCB3 proteins deliver peptides to the endoplasmic reticulum to form class I major histocompatibility complex (MHC) molecules [34], which controls a major part of the immune system in all vertebrates. MHCs display antigens on the cell surface for recognition by the appropriate T-cells. Thus, *ABCB2* and *ABCB3* are link to gnathostomes adaptive immune system. The timing of ABCB2 and ABCB3 appearance parallels the emergence of an adaptive immune system in gnathostomes. In lampreys, two additional *ABCB1*-like and *ABCB10*-like genes, named based on the highest similarity of their encoded proteins to the ABCB1 and ABCB10 proteins of mouse in BLASTP searches, were not well resolved in the phylogenetic analyses.

### ABCC subfamily

**Fig. 3:**
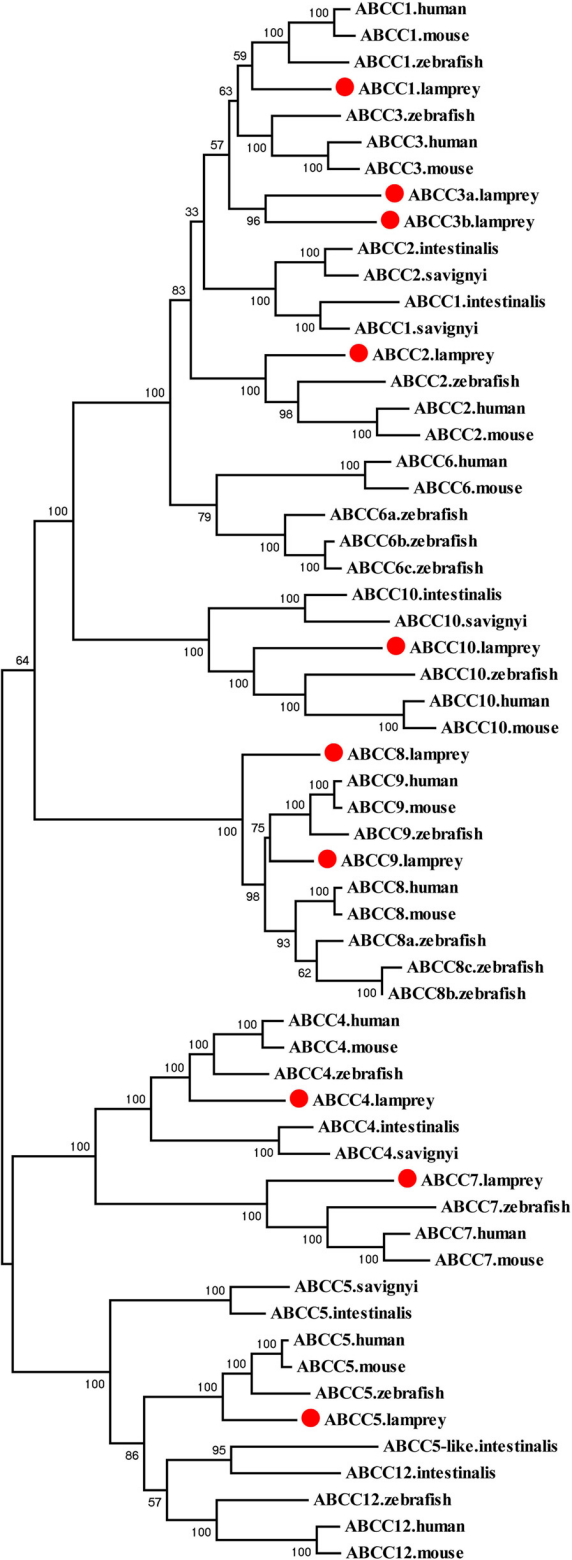
Phylogenetic tree of ABCC subfamily transporters. The phylogenetic tree was constructed as described in the legend to Fig. 1. The lamprey ABCC proteins are highlighted with red dots

Ten ABCC genes were identified in lampreys, including *ABCC1*, *ABCC2*, *ABCC3a*, ABCC3b, *ABCC4*, *ABCC5*, and *ABCC7*–*ABCC10*, all of which encode full ABC transporters (Additional file 5: Figure S3). Only five of the 10 ABCC subfamily members were found in the previous study by Liu et al [22]. Moreover, the *ABCC7* fragment was mistakenly annotated as “*ABCC4*”, the short fragment of *ABCC9* was mistakenly annotated as “*ABCC8-2*”, and *ABCC10* was fragmented into two segments that were annotated as “*ABCC10-1*” and “*ABCC10-2*” (Additional file 3: Table S1). Of the 12 ABCC members in human, *ABCC6*, *ABCC11*, and *ABCC12* were not found in the lampreys. In general, the phylogenetic analyses supported the names assigned to the ABCC genes in the lampreys (Fig. 3). Most lamprey ABCC proteins were positioned at the base of their respective clades with orthologous proteins from other species. Sea squirt ABCC1 and ABCC2 did not form separate clusters with orthologous genes from other species, but clustered with each other and then grouped with the subclade of ABCC1 and ABCC3 from other species. No *ABCC3* was found in sea squirts, but it was exclusively duplicated in lampreys. No *ABCC6* was identified in either sea squirts or lampreys. However, three copies of *ABCC6* were found in the zebrafish genome. Inferred from the phylogenetic tree, *ABCC1* and *ABCC2* in sea squirts may be the ancestral genes of *ABCC1*–*ABCC3* and *ABCC6*; *ABCC6* appeared after lampreys split with teleosts.

Three zebrafish ABCC8 proteins first grouped with human and mouse ABCC8s and then clustered with the ABCC9 subclade. Lamprey ABCC8 was placed at the root of the clade. No *ABCC8* or *ABCC9* genes were identified in sea squirts. These results suggested that *ABCC8* and *ABCC9* may have appeared after the speciation of sea squirts but before the speciation of lampreys. Sea squirt ABCC5 is positioned at the base of the clade that includes ABCC5 and ABCC12 proteins from all the species, except for lamprey ABCC12. *ABCC12* may have been derived from the duplication of *ABCC5* and then underwent an independent gene loss in lampreys, or it was simply not found in the current incomplete lamprey draft genomes.

*ABCC4* was closely related to *ABCC7*, which is not found in sea squirts but present in lampreys. Both *ABCC11* and *ABCC13* were not present in lampreys. *ABCC11* was found to have been duplicated tandemly from *ABCC12* in human [35]. *ABCC13* is present in zebrafish and is still functionally present in chicken, dog, and macaque, but is inactive in human, great apes, and mouse [36].

### ABCD subfamily

**Fig. 4:**
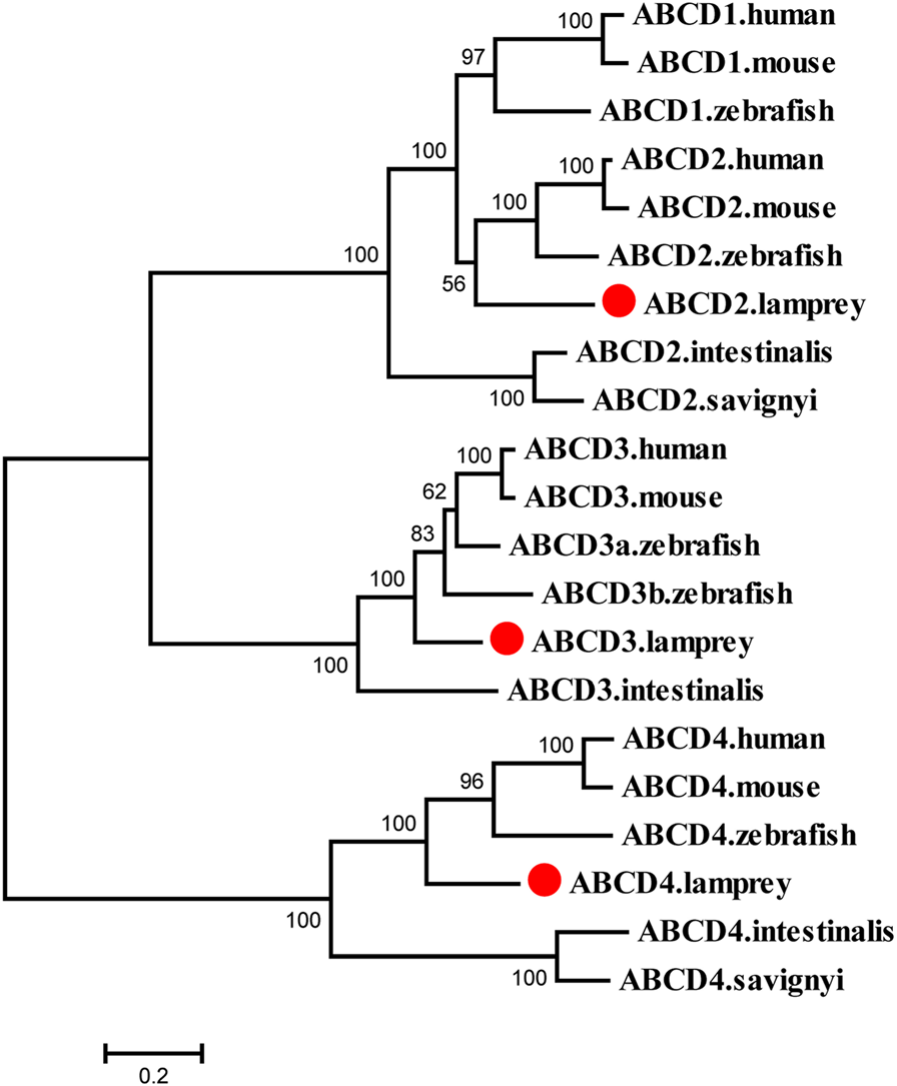
Phylogenetic tree of ABCD subfamily transporters. The phylogenetic tree was constructed as described in the legend to Fig. 1. The lamprey ABCD proteins are highlighted with red dots

All members of the ABCD subfamily are half transporters located in the peroxisome with one TMD and one NBD [8]. All the genes in this subfamily are highly conserved in vertebrates and have undergone very few duplication or loss events. Three out of the four members of ABCD subfamily, *ABCD2*–*ABCD4*, were identified in both sea squirts and lampreys; *ABCD3* was not found by Liu et al (Additional file 3: Table S1). The phylogenetic tree supports the assignment of ABCD2, ABCD3, and ABCD4 in lampreys (Fig. 4). *ABCD1* was not identified in either lampreys or sea squirts. The ABCD1 subclade clustered with the ABCD2 proteins of human, mouse, zebrafish, and lamprey, with the sea squirt ABCD2 positioned at the base of the clade. The phylogenetic tree indicates that *ABCD1* originated from the duplication of *ABCD2* before the divergence of lamprey with gnathostome and then underwent an independent gene loss in the lamprey lineage. Alternatively, *ABCD1* is present in the lamprey genome but was not found in the current incomplete lamprey genome assemblies. *ABCD3* had a single copy in lampreys, mouse, and human; but underwent duplication in fish-specific lineage including zebrafish and catfish [22].

### ABCE and ABCF subfamilies

**Fig. 5:**
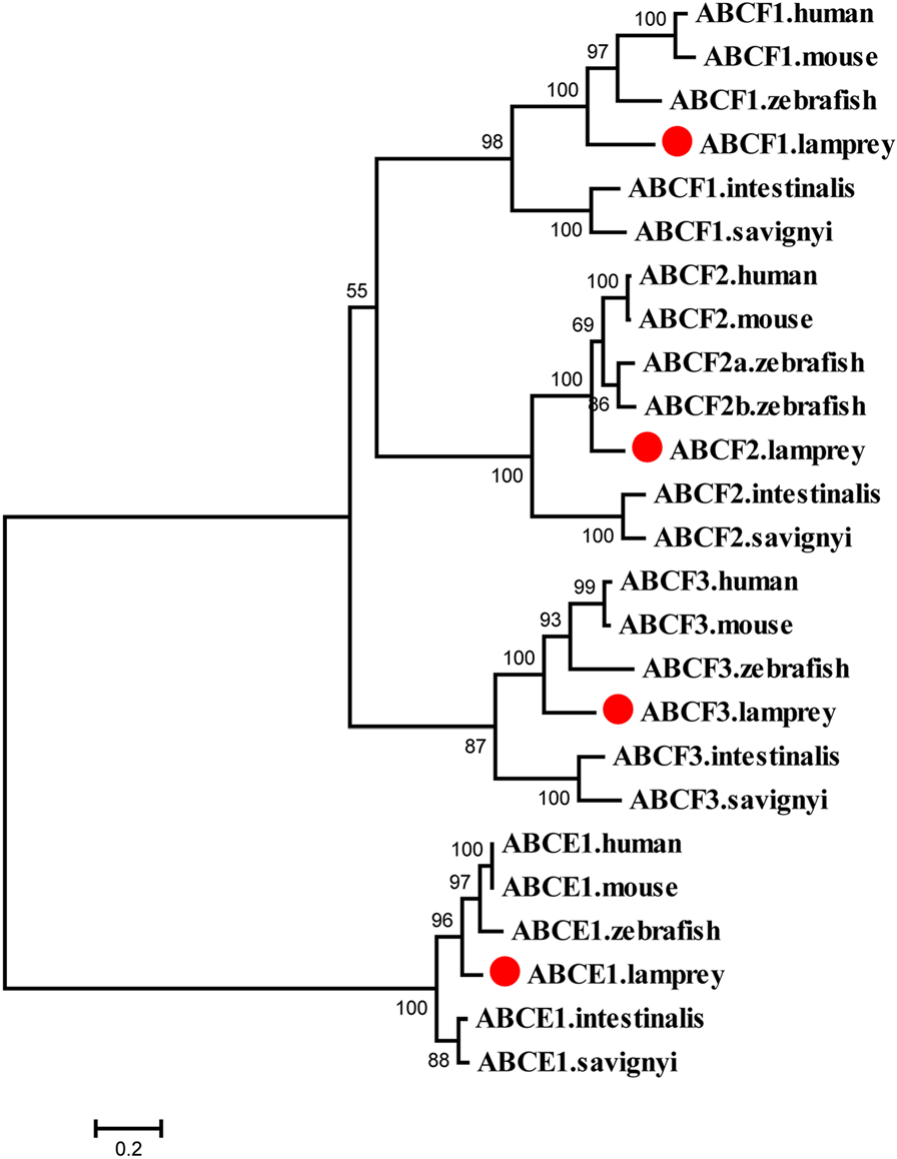
Phylogenetic tree of ABCE and ABCF subfamily transporters. The phylogenetic tree was constructed as described in the legend to Fig. 1. The lamprey ABCE and ABCF proteins are highlighted with red dots

The ABCE and ABCF subfamilies consist of genes containing two NBDs with no TMDs (Table 1, Additional file 5: Figure S3). Members of the ABCE and ABCF subfamilies were highly conserved in chordates; and each of the members identified in sea squirts was positioned at the base of the corresponding subclade. All the ABCE and ABCF members were identified by Liu et al, but *ABCF3* was split into two fragments and named “*ABCF3-1*” and “*ABCF3-2*” (Additional file 3: Table S1). The phylogenetic analysis supported the gene name assignment in lamprey. Each member of the ABCE and ABCF subfamilies was present as a single-copy gene in most species. However, in some teleost fish, the members possessed more copies as a result of gene duplication; for example, there are two *ABCF2* genes in zebrafish (Fig. 5), and two *ABCE1* and *ABCF2* genes in catfish [22].

### ABCG subfamily

**Fig. 6:**
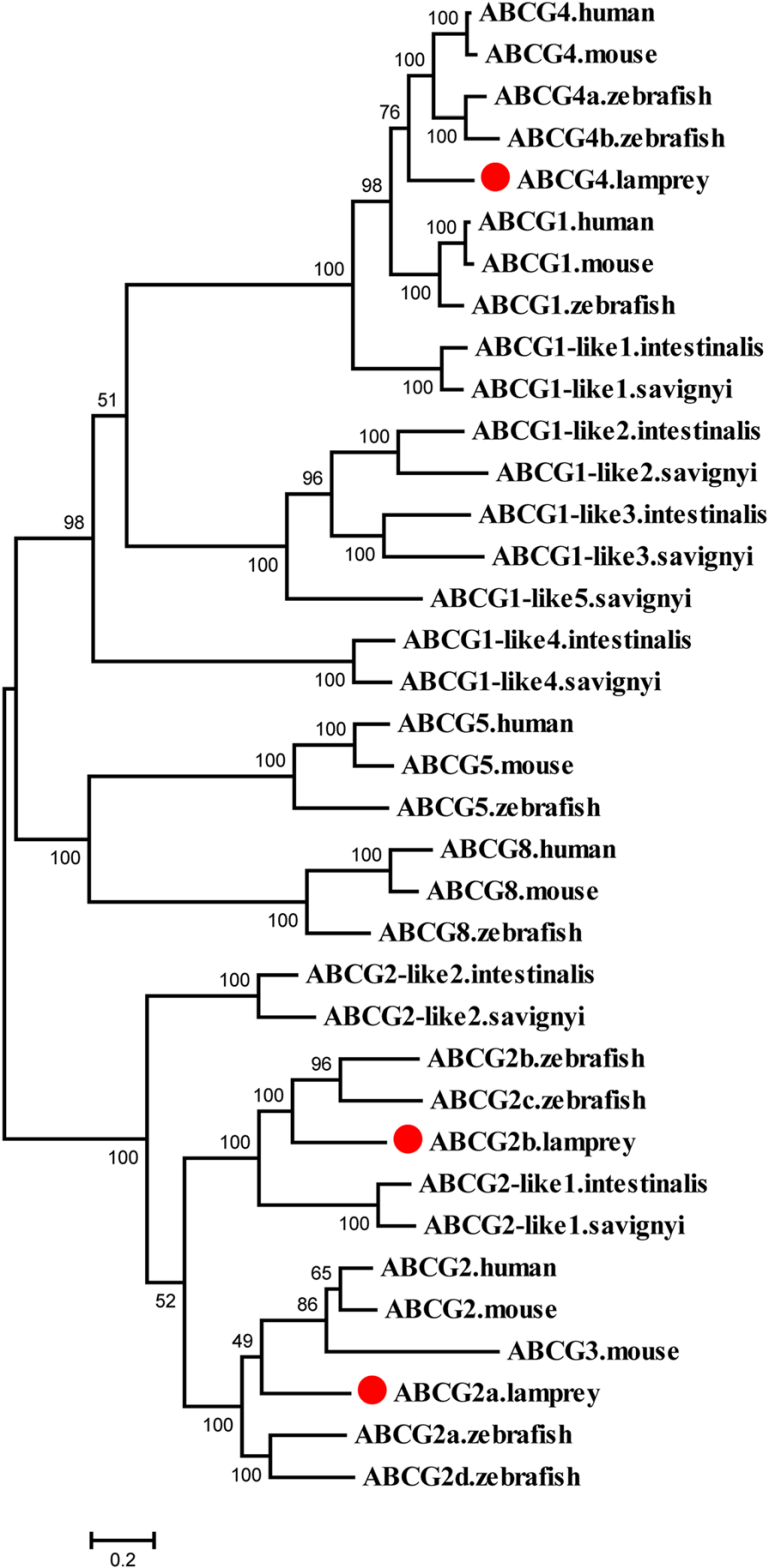
Phylogenetic tree of ABCG subfamily transporters. The phylogenetic tree was constructed as described in the legend to Fig. 1. The lamprey ABCG proteins are highlighted with red dots

All the ABCG transporters in metazoans are half transporters. Unlike other half transporters, the ABCGs have a reverse domain structure with a NBD at the N-terminus and a TMD at the C-terminus. Only three members, *ABCG2a*, *ABCG2b*, and *ABCG4* were found in lampreys, and *ABCG2b* was missed by Liu et al (Additional file 3: Table S1). In human, mouse, and zebrafish, the ABCG subfamily contains at least five members, *ABCG1*, *ABCG2*, *ABCG4*, *ABCG5*, and *ABCG8*. In addition, *ABCG3* was present in mouse, and four copies of *ABCG2* and two copies of *ABCG4* were present in zebrafish (Fig. 6).

Notably, five *ABCG1*-like genes were identified in sea squirts, which are rooted at the clade composed of the ABCG1 and ABCG4 subclades (Fig. 6). These data suggested that *ABCG4* was derived from the duplication of the *ABCG1*-like1 gene before the speciation of lampreys and the remaining *ABCG1*-like genes have undergone gene loss during vertebrate evolution. *ABCG1* was not found in lamprey, suggesting that this gene may have been lost independently in the lamprey lineage or was not found because of the incomplete lamprey genome assembly.

*ABCG5* and *ABCG8* arose from a common ancestral gene as evidenced from the head-to-head arrangement on a chromosome and the encoded proteins form a functional heterodimer [37]. The ABCG5 and ABCG8 proteins clustered together in the phylogenetic tree without orthologs in the lampreys and sea squirts. Two *ABCG2* members were identified in both sea squirts and lampreys and four *ABCG2* were present in zebrafish. The ABCG2 proteins in both lamprey and zebrafish were separated into two subclades; one ABCG2 in lampreys and two ABCG2s in zebrafish formed one subclade with the ABCG2-like1 gene in sea squirts. The ABCG2s in human, mouse, lamprey, and zebrafish clustered with mouse ABCG3 in the other subclade. Furthermore, the ABCG2-like2 protein in sea squirts was placed at the base of the clade (Fig. 6). This result implies that the *ABCG2* genes derived from the *ABCG2*-like1 in sea squirts underwent gene loss in the lineages after the divergence of teleost from other vertebrates. Conversely, the *ABCG2* genes derived from *ABCG2*-like2 in sea squirts remained and duplicated to give rise to *ABCG3* in mouse.

### Expression profiling of ABC genes in tissues across different developmental stages

**Fig. 7:**
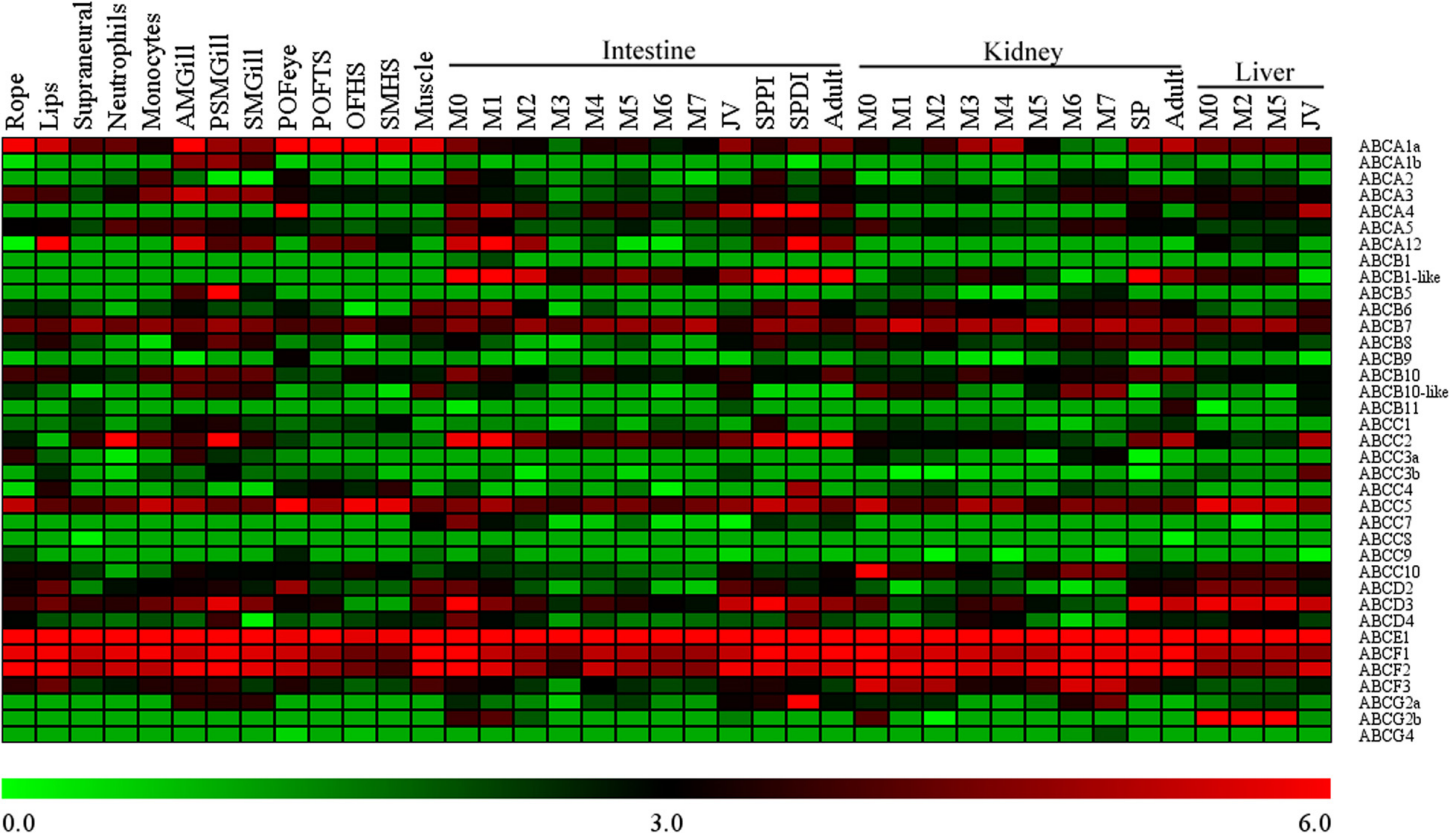
Heat map of expression levels of 37 ABC genes of sea lamprey. Gene expression levels in various tissues across different developmental stages are shown. A colored scale at the bottom of the figure shows the corresponding reads per million (RPM) log2 values. Gene expression levels are shown after log2 data transformation

The expression levels of ABC genes were determined in sea lamprey tissues, including mature male rope, adult lips, supraneural, neutrophils, monocytes, and adult male gill (AMGill), and in tissues at different reproductive stages including prespermiating male gill (PSMGill), spermiating male gill (SMGill), preovulatory female eye (POFEye), preovulatory female tail skin (POFTS), ovulatory female head skin (OFHS), spermiating male head skin (SMHS), and spermiating male muscle (muscle). In addition, the expression levels of ABC genes in the intestine, kidney, and liver tissues across different developmental stages including larvae (M0), metamorphosis (M1-M7), juvenile, parasite ,and adult stages were analyzed (Fig. 7, Additional file 6: Table S2).

The RNA-Seq data showed that all 37 ABC genes were expressed (RPM >1) in at least one of the tissues (Additional file 6: Table S2). The ABCE and ABCF subfamily genes were ubiquitously and highly expressed in all the tested tissues. *ABCB7* and *ABCC5* were also ubiquitously and highly expressed, implying that these genes may play important roles in biological process in sea lamprey. Other genes were differentially expressed across various tissues. Within the same tissue, most of the ABC genes were highly expressed at the larval stage and stages after metamorphosis, but relatively lowly expressed at metamorphosis stages.

Extensive functional studies of the ABC genes have been performed in human and mouse, but few studies have been carried out in lampreys [38, 39]. ABCA1 and ABCA4 are best studied members of the ABCA subfamily. In mammals, the ABCA1 protein is required for cholesterol efflux transport from peripheral cells into high-density lipoproteins particles [40, 41]. The lamprey possessed two orthologs of *ABCA1*, *ABCA1a*, and *ABCA1b. ABCA1a* was ubiquitously and highly expressed in all tissues, especially in the tissues at the adult and mature stages, whereas *ABCA1b* was highly expressed in the gill tissue (13–27 RPM). The ABCA4 protein, also known as photoreceptor rim protein (RmP) or ABCR, was expressed exclusively in the retina and is localized in the outer segment disk edges of rod photoreceptors [42]. Indeed, it is highly expressed in POFEye (162 RPM) and in intestine at M0 and M1 (24 and 38 RPM), and is especially highly expressed in the small parasite stages (261 and 773 RPM). This finding implies that the ABCA4 protein may perform multiple functions in early vertebrates and may have evolved to perform retina-specific function in mammals (human, mouse, and rodent) [43]. The ABCA12 protein appears to be essential for normal development of the skin. Several mutations in this gene have been reported to lead to severe skin disorders such as harlequin ichthyosis and lamellar ichthyosis type 2 in human [44, 45]. Although the exact function of ABCA12 is unknown, it probably plays an important role in transporting lipids into the cells that make up the epidermis [46]. ABCA12 is highly expressed in lip (134 RPM), adult gill (47 RPM), and intestines at M0 and M1 stages (42 and 76 RPM). It is also differentially expressed in different intestine regions at the small parasitic stage (small parasite distal intestine (SPDI), 293 RPM; and small parasite proximal intestine (SPPI), 18 RPM).

ABCB1 (P-gp or MDR1) functions in exporting foreign substances out of cells. The lamprey genomes contain two members of the *ABCB1* gene, *ABCB1* and *ABCB1*-like. *ABCB1* showed no or low expression in all the tissues tested, whereas *ABCB1*-like was highly expressed in intestine at the early stages of metamorphosis, M0–M2 (47–123 RPM), and at the small parasite and adult stages (63–130 RPM), as well as in kidney at the small parasite and adult stages (64 and 25 RPM). In human, *ABCB5* was expressed mainly in normal skin and malignant melanoma [47], while *ABCB5* was expressed in lamprey kidney and gill, with the highest expression level (80 RPM) in PSMGill.

The four half transporters, ABCB6– ABCB8 and ABCB10, were localized in mitochondria where they were reported to function in iron metabolism and transport of Fe/S protein precursors [48]. *ABCB6* was ubiquitously expressed (more than 1 RPM) in all tissues and highly expressed in muscle (19 RPM) and in intestines at the early metamorphic stages M0 and M1 (22 and 27 RPM), as well as in the small parasite stage (14 and 25 RPM). *ABCB7* was also ubiquitously expressed (more than 10 RPM) in all tissues. *ABCB8* was highly expressed (more than 10 RPM) in lips, gills, SPPI, and kidneys at metamorphic stages of M0, M6, and M7, and at the small parasite and adult stages. *ABCB10* was highly expressed in gills (18–19 RPM), in intestine at stages of M0 (23 RPM), and adult (17 RPM), and in kidney at the small parasite and adult stages (17 and 20 RPM). The expression pattern of *ABCB10*-like was different from that of *ABCB10*. In addition, *ABCB10*-like was highly expressed in muscle and kidneys at metamorphic stages. ABCB11 (bile salt export pump, BSEP), is responsible for the transport of taurocholate and other cholate conjugates from hepatocytes to the bile. However, *ABCB11* was not expressed specifically in liver, but was also expressed in kidney [49] and, in general, we found that *ABCB11* was expressed at very low levels in most tissues, but was highly expressed in adult kidney and juvenile liver (12 and 7 RPM). This may be a compensatory mechanism in post-metamorphic lamprey that have lost the biliary tree [50].

*ABCC1* was expressed at low levels in all tissues; its highest expression levels were in SPPI and gills (about 10 RPM). *ABCC2* was expressed at relative high levels in all the tested tissues, and was especially high in neutrophils (67 RPM), PSMGill (150 RPM), and in intestines at stages of M0, M1, small parasite, and adult (52–67 RPM). In addition, *ABCC2* was highly expressed in kidney and liver after metamorphosis. Two copies of *ABCC3*, *ABCC3a*, and *ABCC3b* in lampreys were lowly expressed in all tissues. The highest expression levels (more than 10 RPM) of these genes were in rope, gill, and juvenile liver. *ABCC4* was highly expressed in lips (11 RPM) and SMHS (12 RPM), and differentially expressed in different intestine regions at the small parasite stages (SPPI 0.1 RPM, and SPDI 29 RPM). *ABCC5* was ubiquitously and highly expressed in all tissues with the highest level in POFEye (106 RPM). *ABCC7*/*CFTR* encodes an ion channel that transports chloride and thiocyanate ions across epithelial cell membranes [51, 52]. Its expression levels were generally low in all tissues except for larval intestine (22 RPM). ABCC8 and ABCC9 function as modulators of ATP-sensitive potassium channels and for insulin release [53]. The expression levels of both *ABCC8* and *ABCC9* were generally low in all tissues. *ABCC10*, which encodes a protein whose functions are unclear, was highly expressed in all tissues, especially in larval kidney (61 RPM).

ABCD homodimers are involved in the transport of very long chain acyl-CoA into the peroxisome [54]. *ABCD2* was broadly expressed and its expression was especially high in lips (19 RPM), POFEye (28 RPM), muscle (18 RPM), and intestine at larva (18 RPM) and juvenile (17 RPM) stages. Its expression was relatively low in intestine and kidney at metamorphosis stages, but higher in liver at larval and metamorphic stages (18–21 RPM). *ABCD3* is highly expressed in gill, intestine, and kidney at larval and post-metamorphic stages and in liver at all stages. The expression of *ABCD4* was generally low in all tissues, but in PSMGill and intestine its expression was more than 10 RPM at larva and parasitic stages.

*ABCE1*, ABCF1, and ABCF2 were ubiquitously and highly expressed in all tissues. The gene expression patterns were consistent with the fundamental and essential functions of the encoded proteins in ribosome biogenesis and translation regulation [9–11, 19]. The highest expression levels of *ABCE1* were found in adult intestine and kidney (368 and 347 RPM, respectively). *ABCF3* was also ubiquitously expressed, but its expression was generally lower compared with *ABCE1*, and ABCF1, and ABCF2.

The lamprey genomes contained two copies of *ABCG2. ABCG2a* was highly expressed in gills and kidney at M6 and M7 stages and the highest expression was found in intestine (SPDI 107 RPM). *ABCG2b* showed high expression in intestine and kidney at larval stage and the highest levels were found in liver at larval and metamorphic stages (109 and 132 RPM). *ABCG4* was not expressed in all the tissues.

## Conclusions

The first comprehensive analysis of ABC transporter genes were carried out in a primitive vertebrate lineage, the lampreys. A total of 37 ABC transporter genes were identified. Thirteen more ABC transporter genes were identified compared with a previous study. Through the concatenation of the gene sequences from two lamprey species, full length ABC transporter sequences were obtained, which significantly improved the resolution of the name assignment and phylogenetic position for the genes compared with a previous analysis in which sequence fragments were used. In chordates, the ABC transporter gene subfamilies have undergone obvious expansion or contraction. The ABCA subfamily showed the highest gene expansion rate during chordate evolution. All the genes in the ABCD, ABCE and ABCF subfamilies were highly conserved in the chordates. The evolution of ABC transporters in lampreys needs to be evaluated further because the results presented here are based on a draft genome.

## Methods

### Identification of ABC transporter genes

We used genomic resources, including the nucleotide and protein sequences of the sea lamprey and Japanese lamprey genomes, together with a sea lamprey transcriptome assembly of 89 RNA-Seq samples from various tissues across different developmental stages (unpublished data, Additional file 1: Figure S1), to identify lamprey ABC transporter genes. All available ABC transporter proteins of human, mouse and zebrafish were retrieved from GenBank or the Ensembl genome browser (release75) [55, 56] (Additional file 7: Table S3) and used as queries in standalone BLASTP or TBLASTN searches against the lamprey genomic resources. The coding regions in the retrieved RNA-Seq transcripts were predicted using the GETORF program in the EMBOSS online tool (EMBOSS GUI v1.14; http://imed.med.ucm.es/cgi-bin/emboss.pl?_action=input&_app=getorf) [57], and the retrieved genomic sequences were subjected to *ab initio* gene prediction using the Augustus program (http://bioinf.uni-greifswald.de/augustus/) [58] and protein-based similarity gene prediction using FGENESH+ web server (http://www.softberry.com/) [59]. The final protein-coding sequences were validated by using them as queries in BLASTP searches against the NCBI non-redundant protein sequence database (nr). The conserved domains of the identified ABC proteins were predicted using SMART (simple modular architecture research tool) [60] and confirmed using the NCBI CD-Search tool to predict conserved domains.

### Phylogenetic analysis

All available ABC transporter proteins of human, mouse, zebrafish, and two sea squirts (*C. intestinalis* and *C. savignyi*) were retrieved from GenBank or the Ensembl genome browser (release75), and used for the phylogenetic analyses (Additional file 7: Table S3). The ABC transporter genes of *C. intestinalis* have been reported previously based on the genome assembly v1.0 at the Joint Genome Institute (JGI; http://genome.jgi-psf.org) [23]. Therefore, we downloaded the ABC transporter proteins of *C. intestinalis* from JGI and used them as queries in BLASTP searches against the protein sequences of *C. intestinalis* and *C. savignyi* from Ensembl. The three ABCC subfamily members that were present in the JGI genome assembly v1.0 but were not in the Ensembl genome were included in the phylogenetic analysis.

Several of the ABC genes that were identified in both lamprey genomes were partial sequences. To compare the lamprey ABC sequences with the ABC sequences from the other vertebrate genomes, some of the ABC gene sequences from both lamprey genomes were concatenated to obtain longer sequences for the phylogenetic analyses. The concatenation of sequences from the two lamprey species was feasible because the nucleotide sequences in the protein-coding regions shared similarities of 97 % or more between the two genomes. The concatenated ABC sequences had a negligible effect on the topology of the phylogenetic tree. To improve the phylogenetic resolution, all the ABC transporter proteins were used to construct a tree and then the proteins within each subfamily were used to construct separate trees. The protein sequences for human, mouse, zebrafish, sea squirts, and the two lamprey were aligned using ClustalX 1.83 [61], then the multiple alignments were used to the phylogenetic tree using MEGA 6 [62] with 100 bootstrap replicates. The phylogenetic trees were constructed using the maximum likelihood method with the Jones–Taylor–Thornton (JTT) substitution model. The assignment of the lamprey ABC proteins subfamily member was based on both the phylogenetic analysis and the highest similarity between the lamprey and mouse ABC proteins in the BLASTP searches.

### Quantification of ABC gene expression

The concatenated ABC gene sequences from both lamprey genomes were indexed as the reference sequences with Bowtie 1.0.0 [63]. RNA samples from an assortment of 89 tissues across different developmental stages were extracted using TRIzol method according to the manufacturer’s instructions (Invitrogen, Foster City, CA, USA). The RNA was then poly-A selected, fragmented, and reverse transcribed to cDNA. The cDNA was sequenced using the Illumina GAII platform by the Research Technology Support Facility (RTSF) at Michigan State University using the Illumina mRNA-Seq 8-Sample Prep Kit. About 20 million reads, 75–100 nucleotides long, were obtained per sample giving a total of 1.5–2 Gb of mRNA-Seq sequencing reads. The reads were filtered, then trimmed using Trimmomatic [64], and aligned to the reference sequences using TopHat 1.4.1 with the default parameters [65]. The number of reads mapped to a specific gene was counted based on the BAM file. The gene expression level was calculated as the number of reads mapped to the specific gene relative to the total number of the filtered reads in the sample. The ABC gene expression level was normalized as RPM (the number of reads assigned to a gene per million reads). To better present the gene expression levels in the heat map, the expression values were transformed to log2 data. If the gene expression value was less than 1 RPM, it was assigned to 1 RPM. The gene expression values after the log2 data transformations are shown in the heat map, which was constructed using the MeV v4.0 package [66].

### Availability of supporting data

The original data for phylogenetic analysis can be accessed on Dryad: doi:10.5061/dryad.k867f. The sea lamprey ABC gene sequences have been deposited in GenBank database (accession numbers: KM232912–KM232947) whereas the Japanese lamprey ABC gene sequences are included within the Additional file 2.

## Additional files

Additional file 1: Figure S1. Tissues across developmental stages for mRNA-Seq. Additional file 2:
**Sequences of ABC transporter genes identified in Japanese lamprey.**
Additional file 3: Table S1. Comparison of the lamprey ABC transporter genes between the Ensembl sequences and the sequences obtained in this study. Additional file 4: Figure S2. Phylogenetic tree of all the ABC transporter proteins used in this study. Additional file 5: Figure S3. Functional domain organization in 37 lamprey ABC transporters. Additional file 6: Table S2. Tissues and expression values of ABC genes in sea lamprey. Additional file 7: Table S3. All ABC transporter proteins retrieved and used in this study.

## Abbreviations

ABC: ATP-binding cassette
TMD: Transmembrane domain
NBD: Nucleotide-binding domain
MDR: Multidrug resistance protein
MRP: Multidrug resistance associated protein
P-gp: P-glycoprotein
TAP: Transporter associated with antigen processing
CFTR: Cystic fibrosis transmembrane conductance regulator
ALD: Adrenoleukodystrophy
BSEP: Bile salt export pump
RPM: Reads per million mapped reads
AMGill: Adult male gill
PSMGill: Prespermiating male gill
SMGill: Spermiating male gill
POFEye: Preovulatory female eye
POFTS: Preovulatory female tail skin
OFHS: Ovulatory female head skin
SMHS: Spermiating male head skin
SPPI: Small parasite proximal intestine
SPDI: Small parasite distal intestine
