# Supplementary material for: Genome-wide analysis of the ATP-binding cassette (ABC) transporter gene family in sea lamprey and Japanese lamprey
Source: BMC Genomics. 2015 Jun 6;16(1):436. doi: 10.1186/s12864-015-1677-z (PMC4458048; doi:10.1186/s12864-015-1677-z)
Supplement: Additional file 1: Figure S1. — Tissues across developmental stages for mRNA-Seq. [file 12864_2015_1677_MOESM1_ESM.pptx]

## Slide 1
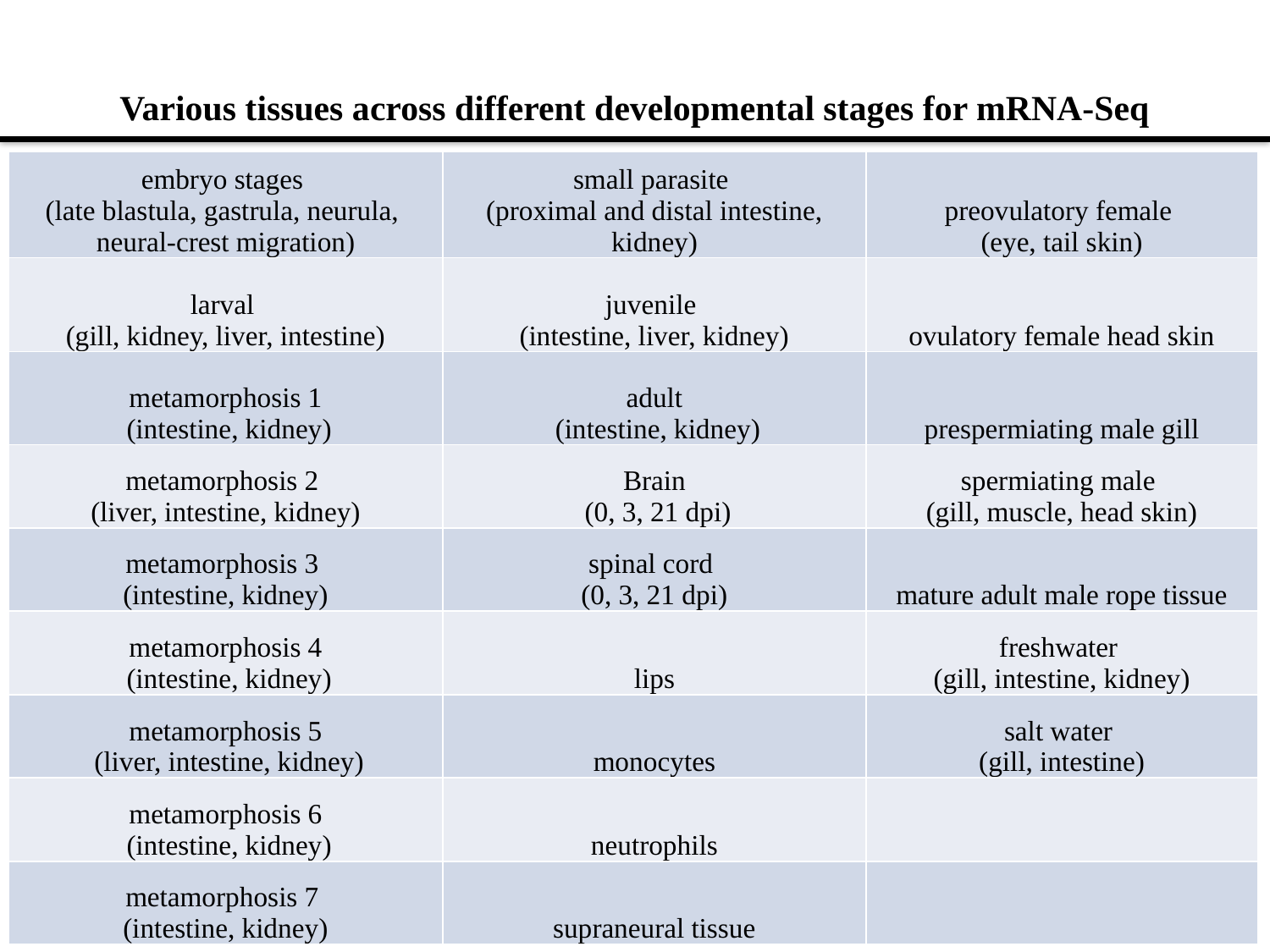

# Various tissues across different developmental stages for mRNA-Seq
| embryo stages (late blastula, gastrula, neurula, neural-crest migration) | small parasite (proximal and distal intestine, kidney) | preovulatory female (eye, tail skin) |
| --- | --- | --- |
| larval (gill, kidney, liver, intestine) | juvenile (intestine, liver, kidney) | ovulatory female head skin |
| metamorphosis 1 (intestine, kidney) | adult (intestine, kidney) | prespermiating male gill |
| metamorphosis 2 (liver, intestine, kidney) | Brain (0, 3, 21 dpi) | spermiating male (gill, muscle, head skin) |
| metamorphosis 3 (intestine, kidney) | spinal cord (0, 3, 21 dpi) | mature adult male rope tissue |
| metamorphosis 4 (intestine, kidney) | lips | freshwater (gill, intestine, kidney) |
| metamorphosis 5 (liver, intestine, kidney) | monocytes | salt water (gill, intestine) |
| metamorphosis 6 (intestine, kidney) | neutrophils | |
| metamorphosis 7 (intestine, kidney) | supraneural tissue | |
