# Supplementary material for: Genome-wide analysis of the ATP-binding cassette (ABC) transporter gene family in sea lamprey and Japanese lamprey
Source: BMC Genomics. 2015 Jun 6;16(1):436. doi: 10.1186/s12864-015-1677-z (PMC4458048; doi:10.1186/s12864-015-1677-z)
Supplement: Additional file 3: Table S1. — Comparison of the lamprey ABC transporter genes between the Ensembl sequences and the sequences obtained in this study. [file 12864_2015_1677_MOESM3_ESM.docx]

Table S1 Comparison of ABC transporter genes identified between Ensembl and this study

| **Gene subfamily** | **Ensembl Sea lamprey (Liu et al, 2013)** | | | **Concatenation of SL and JL (This study)** | | **Notes** |
| --- | --- | --- | --- | --- | --- | --- |
|  | **Gene name** | **Ensembl protein ID** | **Protein length (aa)** | **Gene name** | **Protein length (aa)** |  |
| ABCA | ABCA1 | ENSPMAP00000002483 | 1103 | ABCA1a | 2276 |  |
|  | - | - | - | ABCA1b | 1471 |  |
|  | ABCA2 | ENSPMAP00000005699 | 398 | ABCA2 | 2457 |  |
|  | ABCA3 | ENSPMAP00000010351 | 303 | ABCA3 | 1725 |  |
|  | - | - | - | ABCA4 | 2116 |  |
|  | ABCA5 | ENSPMAP00000006958 | 686 | ABCA5 | 1679 |  |
|  | ABCA12 | ENSPMAP00000005275 | 1781 | ABCA12 | 1832 |  |
| ABCB | ABCB4 | ENSPMAP00000003844 | 1061 | ABCB1 | 1288 | incorrect name assignment in Liu et al |
|  | ABCB5 | ENSPMAP00000009961 | 153 | ABCB1 | 1288 | incorrect name assignment in Liu et al |
|  | - | - | - | ABCB1-like | 1043 |  |
|  | - | - | - | ABCB5 | 1336 |  |
|  | ABCB6 | ENSPMAP00000004737 | 472 | ABCB6 | 857 |  |
|  | - | - | - | ABCB7 | 729 |  |
|  | ABCB8 | ENSPMAP00000004654 | 626 | ABCB8 | 709 |  |
|  | ABCB9 | ENSPMAP00000009247 | 807 | ABCB9 | 807 |  |
|  | ABCB10 | ENSPMAP00000009848 | 598 | ABCB10 | 754 |  |
|  | - | - | - | ABCB10-like | 790 |  |
|  | ABCB11 | ENSPMAP00000006936 | 1307 | ABCB11 | 1320 |  |
| ABCC | - | - | - | ABCC1 | 1501 |  |
|  | - | - | - | ABCC2 | 1558 |  |
|  | ABCC3 | ENSPMAP00000000990 | 1516 | ABCC3a | 1556 |  |
|  | - | - | - | ABCC3b | 1166 |  |
|  | - | - | - | ABCC4 | 1303 |  |
|  | - | - | - | ABCC5 | 1439 |  |
|  | ABCC4 | ENSPMAP00000000991 | 546 | ABCC7 | 1466 | incorrect name assignment in Liu et al |
|  | ABCC8-1 | ENSPMAP00000008931 | 1578 | ABCC8 | 1578 |  |
|  | ABCC8-2 | ENSPMAP00000001529 | 519 | ABCC9 | 1596 | incorrect name assignment in Liu et al |
|  | ABCC10-1 | ENSPMAP00000005164 | 823 | ABCC10 | 1584 | one fragment of ABCC10 |
|  | ABCC10-2 | ENSPMAP00000007045 | 539 | ABCC10 | 1584 | one fragment of ABCC10 |
| ABCD | ABCD2 | ENSPMAP00000010204 | 277 | ABCD2 | 791 |  |
|  | - | - | - | ABCD3 | 659 |  |
|  | ABCD4 | ENSPMAP00000002275 | 595 | ABCD4 | 633 |  |
| ABCE | ABCE1 | ENSPMAP00000009388 | 599 | ABCE1 | 599 |  |
| ABCF | ABCF1 | ENSPMAP00000008110 | 290 | ABCF1 | 828 |  |
|  | ABCF2 | ENSPMAP00000007650 | 612 | ABCF2 | 614 |  |
|  | ABCF3-1 | ENSPMAP00000005058 | 98 | ABCF3 | 712 | one fragment of ABCF3 |
|  | ABCF3-2 | ENSPMAP00000009514 | 281 | ABCF3 | 712 | one fragment of ABCF3 |
| ABCG | ABCG2 | ENSPMAP00000007015 | 669 | ABCG2a | 703 |  |
|  | - | - | - | ABCG2b | 666 |  |
|  | ABCG4 | ENSPMAP00000001172 | 233 | ABCG4 | 658 |  |
