# Supplementary figures and images for: Genome-wide analysis of the ATP-binding cassette (ABC) transporter gene family in sea lamprey and Japanese lamprey
Source: BMC Genomics. 2015 Jun 6;16(1):436. doi: 10.1186/s12864-015-1677-z (PMC4458048; doi:10.1186/s12864-015-1677-z)

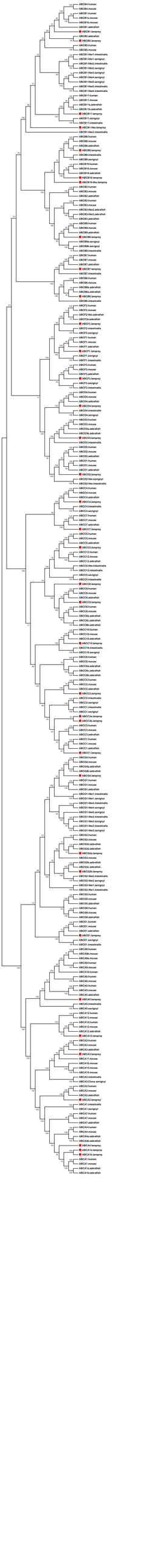

Supplement: Additional file 4: Figure S2. — Phylogenetic tree of all the ABC transporter proteins used in this study. [file 12864_2015_1677_MOESM4_ESM.pdf]
