# Supplementary material for: Genome-wide analysis of the ATP-binding cassette (ABC) transporter gene family in sea lamprey and Japanese lamprey
Source: BMC Genomics. 2015 Jun 6;16(1):436. doi: 10.1186/s12864-015-1677-z (PMC4458048; doi:10.1186/s12864-015-1677-z)
Supplement: Additional file 5: Figure S3. — Functional domain organization in 37 lamprey ABC transporters. [file 12864_2015_1677_MOESM5_ESM.pdf]

ABCA subfamily:

ABCA1a

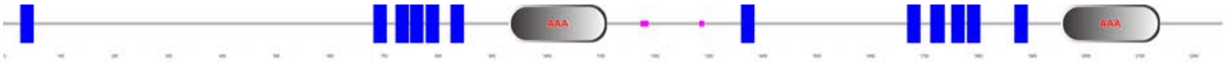

ABCA1b

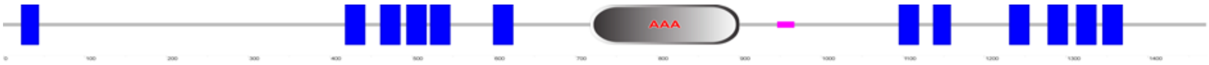

ABCA2

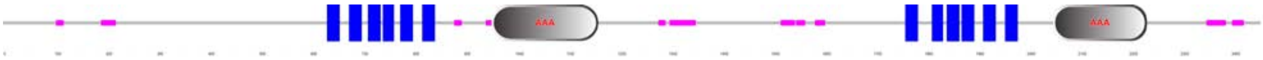

ABCA3

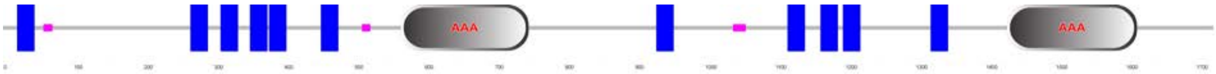

ABCA4

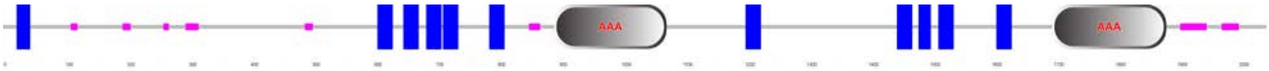

ABCA5

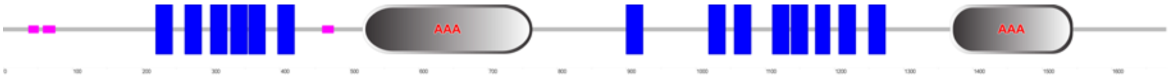

ABCA12

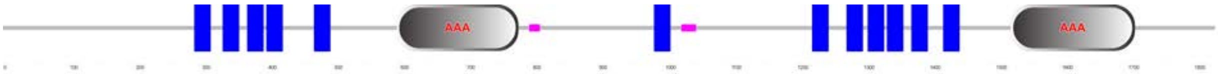

ABCB subfamily:

ABCB1

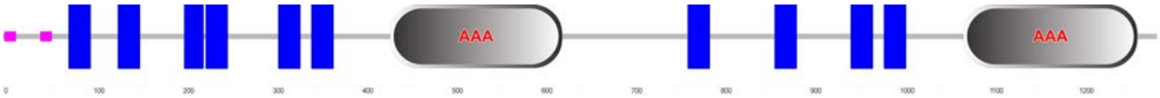

ABCB1-like

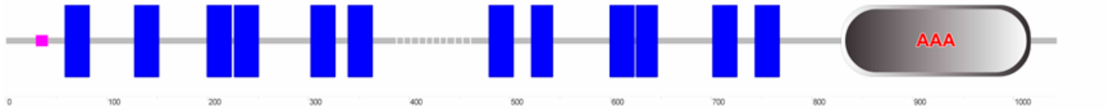

ABCB5

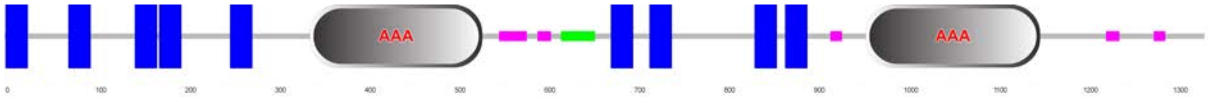

ABCB6

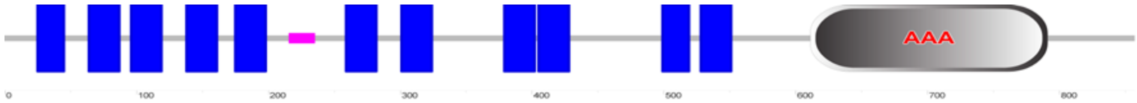

ABCB7

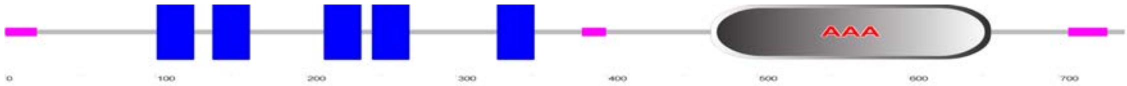

ABCB8

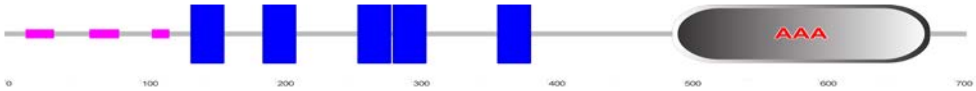

ABCB9

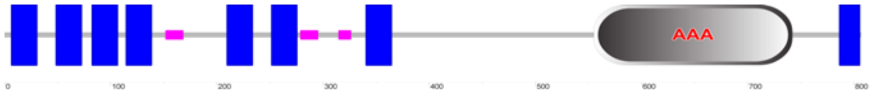

ABCB10

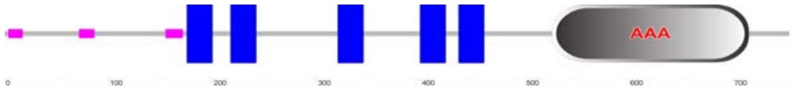

ABCB10-like

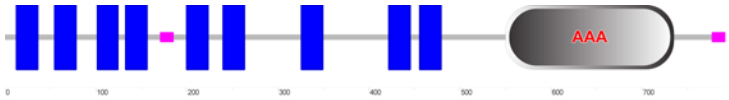

ABCB11

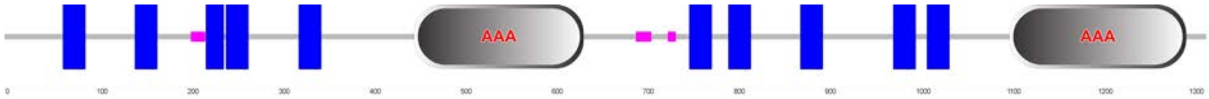

ABCC subfamily:

ABCC1

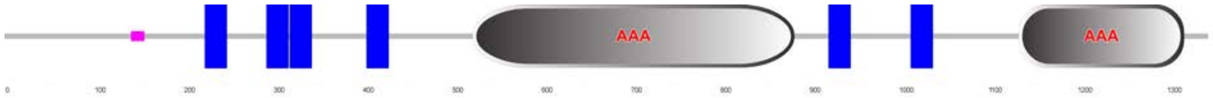

ABCC2

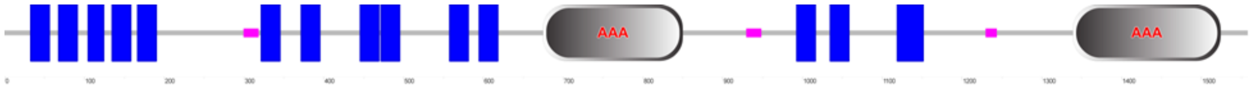

ABCC3a

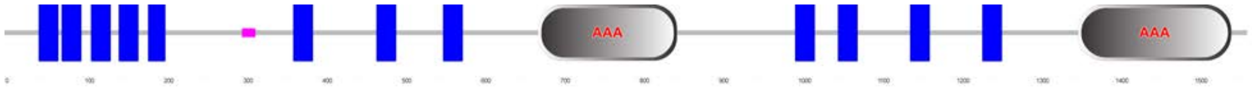

ABCC3b

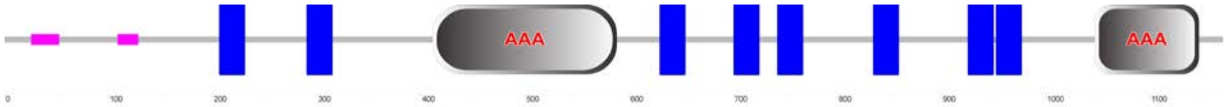

ABCC4

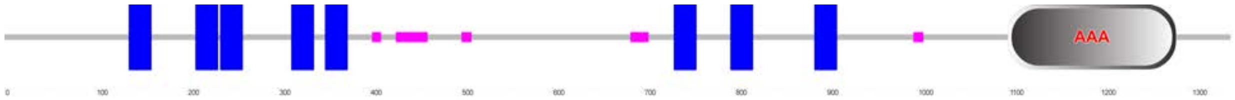

ABCC5

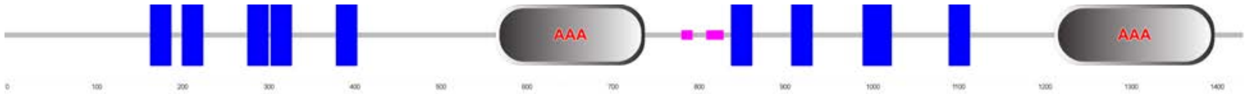

ABCC7

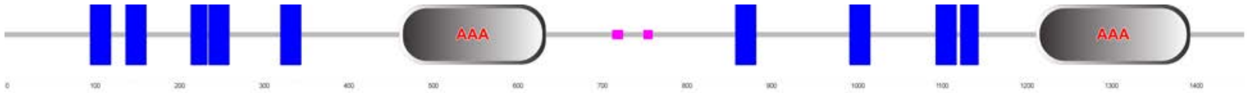

ABCC8

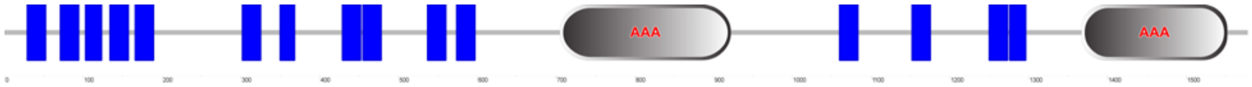

ABCC9

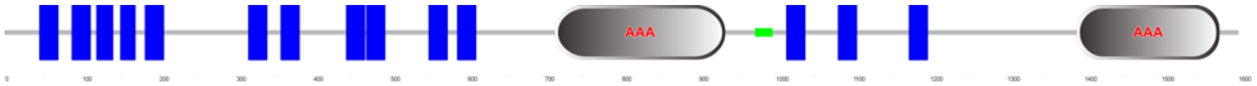

ABCC10

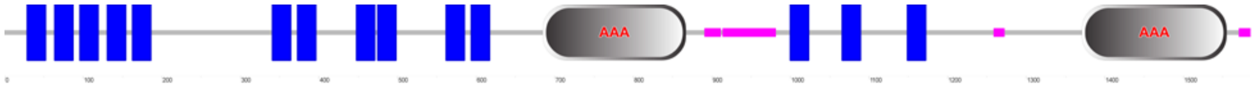

ABCD subfamily:

ABCD2

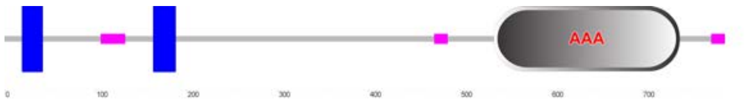

ABCD3

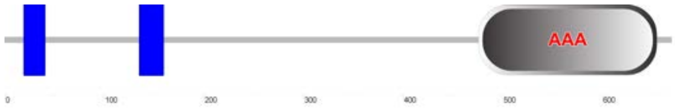

ABCD4

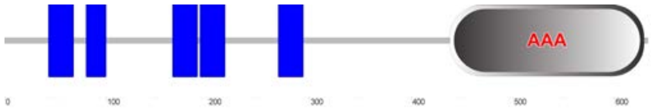

ABCE subfamily:

ABCE1

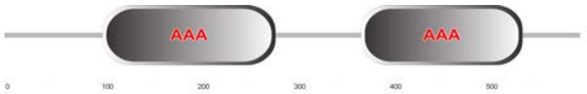

ABCF subfamily:

ABCE1

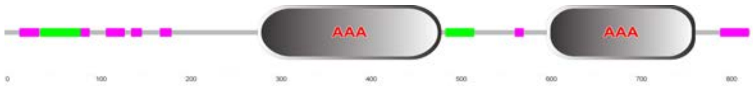

ABCF2

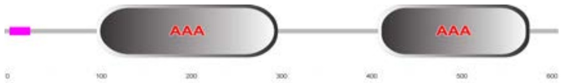

ABCF3

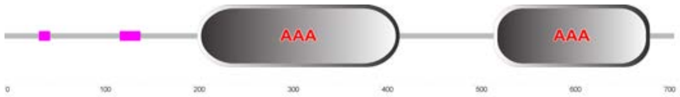

ABCG subfamily:

ABCG2a

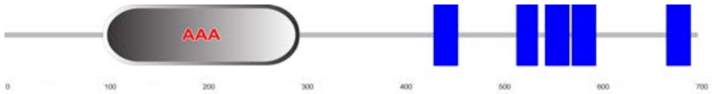

ABCG2b

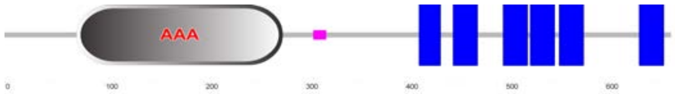

ABCG4

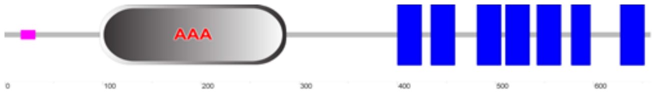

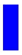 **Transmembrane Domain** 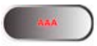 **Nucleotide Binding Domain**
